# Boosting the photocatalytic activity of ZnO-NPs through the incorporation of C-dot and preparation of nanocomposite materials

**DOI:** 10.1016/j.heliyon.2023.e20717

**Published:** 2023-10-05

**Authors:** Asegid Belete Tegenaw, Ahmed Awol Yimer, Tamene Tadesse Beyene

**Affiliations:** Department of Chemistry, College of Natural Sciences, Jimma University, P.O.Box 378, Jimma, Ethiopia

**Keywords:** Nanocomposite, Photocatalytic degradation, Energy band gap, Methylene blue dye, Surface charge, Carbon dot, ZnO-NPs

## Abstract

Due to their applications in cosmetology, medicine, antibacterial and other fields, zinc oxide nanoparticles (ZnO-NPs) are among the nanoscale materials experiencing exponential growth. In contrast, pure ZnO-NPs have been reported to have a very large energy bandgap, a large exaction binding energy, electron-hole recombination, no visible light absorption, and poor photocatalytic activities, which limit their potential uses. ZnO-NPs can be further extended through the incorporation of trace amounts of carbon materials to engulf these problems. We investigate the photocatalytic degradation of methylene blue (MB) dye with pure ZnO-NPs infused with a limited amount of carbon dot (C-dot) materials. Consequently, adding 10% C-dot to ZnO-NPs reduced their energy bandgap from 3.1 to 2.8 eV and significantly increased their photocatalytic activity. MB was almost completely degraded (98.4%) after 60 min when 50 mg of C-dot-incorporated ZnO-NPs were added. By comparison, the nanocomposite's photocatalytic activity exceeded that of pure ZnO-NPs by more than 50%. A surface charge and stability improvement are responsible for the extraordinary photocatalytic improvement. As far as we know, this is the best-ever photocatalytic improvement achieved by incorporating a trace amount of C-dot material into pure ZnO-NPs.

## Highlights

• Incorporation of C-dot brought good performance.
• C-dot improved surface charge and reduced the energy band gap of ZnO-NPs.
• Improved surface charge and a reduced energy band gap resulted in enhanced photocatalytic degradation efficiency.
• MB dye was completely decolourized and >98.4%% optimized degradation efficiency was achieved for the nanocomposite after an hour.

## Graphical abstract

**Figure:**
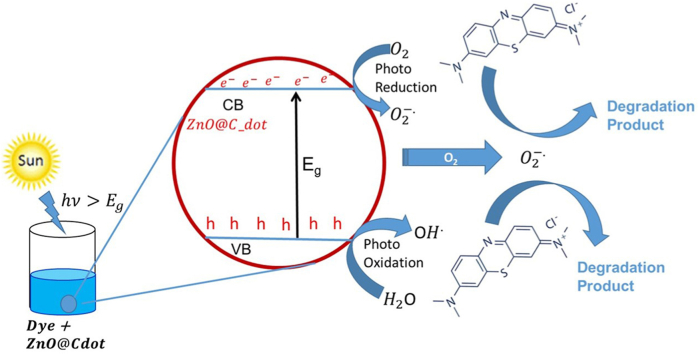

## 1 Introduction

Nanotechnology focuses on studying materials at the nano-scale (1–100 nm). The discovery of new essential and applied frontiers in material science and engineering has been aided by the field of nanotechnology [[1], [2], [3]]. Metal oxide nanoparticles (MONPs) are versatile nanomaterials that can be used in various applications, such as catalysis, optoelectronic materials, sensors, and environmental remediation. Among the most common MONPs are Ag_2_O, CuO, ZnO, TiO_2_, Al_2_O_3_, CrO_2_, NiO and more [[4], [5], [6]]. Researchers have shown interest in Zinc oxide nanoparticles (ZnO-NPs) for their therapeutic and diagnostic properties [4]. They have low toxicity, are biodegradable, and are cost-effective [3]. ZnO nanoparticles can be safely utilized as medicine, preservatives in packaging, photocatalysts, and antimicrobial agents. This substance readily penetrates food, eliminates harmful microorganisms, and protects humans from getting sick [7]. Moreover, it highly increases the photocatalytic degradations of some persistent dyes that induce severe environmental pollution. However, its wide band gap of ZnO (∼3.37 eV) [[8], [9], [10]], a large exaction binding energy of 60 meV [11,12], and agglomeration [13,14] hinder it from potential applications [3,7]. It's crucial to modify ZnO to optimize sunlight usage and improve optical properties. The physical and chemical properties of ZnO-NPs could be tuned by changing their morphology using various synthesis routes and incorporating different materials [7]. Preparation of ZnO-nanocomposites improves the drawbacks associated with the bare ZnO-NPs. Nanocomposite materials can exhibit individual properties such as mechanical [[15], [16], [17]], chemical, electrical, optical and catalytic properties [4,18,19] which make them preferable compared with their respective individual nanoparticles.

Quasi-zero-dimensional carbon materials like carbon dots (CDs) and their composites are being explored for energy storage and photocatalytic applications [20,21]. CDs offer a large surface area, adjustable heteroatom doping, and functional groups [22,23]. The mentioned properties of C-dots could be taken as advantages to improve the surface functionalities and stabilities of other nanomaterials and, hence, their physical and chemical properties.

Here we report the effect of incorporating carbon dot (C-dot) into ZnO-NPs and the preparation of nanocomposite materials (ZnO@C-dot) on its energy band gap and the kinetics of the photocatalytic degradation of methylene blue dye. Accordingly, the chemical Sol-gel method synthesized ZnO-NPs and their C-dot nanocomposites (ZnO@C-dot) successfully [24,25]. The optical properties of the synthesized nanoparticle and its composites were characterised using the spectrophotometric technique (UV–Vis spectrophotometer) and found that the incorporation of C-dot shifted the maximum absorbance wavelength towards red. Following the absorbance shift, the energy band gap of ZnO-NPs was also reduced from 3.14 to 2.80 eV Due to the reduction in band gap, C-dots incorporated into ZnO-NPs exhibited higher photocatalytic activity than bare ZnO-NPs in degrading methylene blue dye. This study provides considerable band gap reduction, super high photocatalytic performance, and excellent reusability of the ZnO@C-dot nanocomposite compared to the pristine ZnO-NPs.

## 2 Materials and method

Pure zinc oxide nanoparticles were synthesized using the sol-gel method to examine the impact of incorporating C-dots into ZnO. The Sol-gel method is preferred because it offers advantages such as repeatability, control over compositions, and simplicity in processing. Prepared ZnO nanoparticles were mixed with a suitable amount of chemically synthesized C-dot and calcined to obtain the ZnO@C-dot nanocomposite.

### 2.1 Chemicals & instruments

Chemicals such as Zinc Nitrate dihydrate (Zn(NO_3_)_2_)·2H_2_O ≥ 98%, (Loba Chemie Pvt. Ltd), Sodium hydroxide (NaOH) ≥ 98% (Blulux Laboratories Ltd-121,005), Ethanol (CH_2_COOH), Citric acid (HOC(COOH) (CH_2_COOH)_2_), (≥99%), Urea (NH_2_CONH_2_) and double-distilled water were used in this research work.

Different laboratory tools were utilized including beakers, measuring cylinders, magnetic stirrers, electronic balances, muffle furnaces, crystallizing dishes, burettes, funnels, filter paper, pH meters, spoons, sample bottles, and pipettes. Advanced analytical instruments such as XRD (Drywell XRD-7000, Cu Kα (λ = 1.54178 Å) radiation), UV–Vis (SPECORD 200 PLUS - 223E1128F), and FT-IR (PerkinElmer Spectrum 2, wavenumber range 8300–350 cm-1), and SEM were used in this research.

### 2.2 Synthesis of ZnO-NPs and its C-dot nanocomposites

Zinc oxide nanoparticles (ZnO-NPs) were synthesized from zinc nitrate dihydrate (Zn(NO_3_)_2_·2H_2_O) using the sol-gel method. 7 g of zinc nitrate dihydrate (Zn(NO_3_)_2_·2H_2_O) were dissolved in 50 mL of double-distilled water to produce ZnO-NPs. Then, for the next 20 min, the two solutions were continuously stirred as 10 g of sodium hydroxide was dissolved in 10 mL of distilled water. The zinc acetate solution was thoroughly blended before the sodium hydroxide solution was added and stirred for 5 min. The mixture of zinc nitrate and sodium hydroxide was then vigorously stirred while being titrated dropwise with ethanol from a burette. The solution was filtered using Whatman filter paper No. 1, and the precipitate was dried at 80 °C for 8 h. After being dried, the white powder was calcined in a muffle furnace for 3 h at 250 °C and given the name ZnO-NPs [10].

Carbon dots (C-dots) were prepared by mixing urea and citric acid solutions prepared in double-distilled water, as reported in Rufina et al. [26,27]. More specifically, 1.0 g of citric acid and 0.5 g of urea were each dissolved in 25 mL of double-distilled water, and the mixture was then stirred at 800 rpm until the solution turned clear. After that, 5 mL of a 0.1 M NaOH solution was added, and 5 min were spent stirring. The obtained solution was then heated for 2 h at 90 °C in an oven. A blue-black solid that was designated as a C-dot was produced after an immediate drying of the dark brown solution that had been heated for 2 h.

**Fig. 1:**
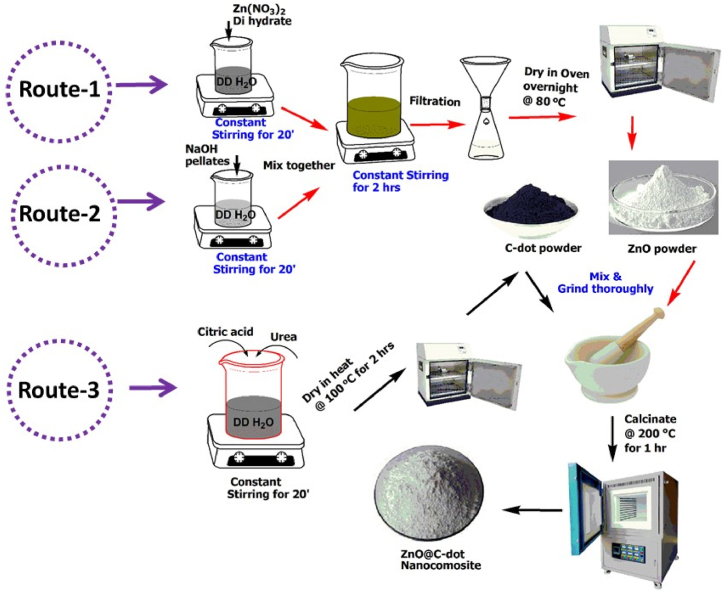
Schematic representation of the synthesis of ZnO-NPs and their composites of C-dot (ZnO@C-dot).

Variations in the ZnO-to-C-dot ratio were used to incorporate C-dot into the ZnO-NPs while the mixture was being calcined at 250 °C. By measuring the composite's UV–Vis absorption based on the absorbance shift, the precise ratios of the two were determined. In Fig. 1, the general synthesis processes of the pure and nanocomposites are shown schematically.

### 2.3 Characterizations

By using XRD, FTIR, and UV–vis spectrophotometers, the prepared samples of pure (ZnO-NPs) and modified ZnO nanoparticles were characterized. 0.2 M solutions in ethanol were used for UV–Vis measurements, while XRD and FT-IR utilized solid powders. Crystal structure and phase composition of synthesized samples were determined using X-ray diffraction (XRD) at a voltage of 40 kV, filament current of 30 mA, and CuKα1 radiation (λ = 1.5418 Å) in the 2θ range of 10°–80°. We used a UV–UV-visible spectrophotometer to calculate the band gap energies and take absorbance measurements. The chemical composition of the samples was examined using Fourier transform infrared (FT-IR) in the wave number range of 400–4000 cm^−1^. Scanning electron microscopy (SEM) was also used to analyse the samples' morphologies and microstructures.

### 2.4 Photocatalytic experiments

The photocatalytic activities of ZnO-NPs and their C-dot nanocomposites were investigated by exposing an MB aqueous solution to direct solar irradiation in January between 11:00 and 14:00. The area's average daily temperature (Jimma) was 30 ± 2 °C degrees. In the supplemental information (Table S1), meteorological data for the study area was given. The solutions were magnetically stirred in the dark for 20 min to achieve adsorption-desorption equilibrium between the photocatalyst and the dye prior to radiation exposure. The photocatalytic degradation of MB was then tested using various concentrations of the photocatalyst in a stock solution of 1000 mg/L of the MB dye in double-distilled water. Based on UV–vis measurement at the proper wavelength, the removal efficiency of MB was calculated.

The point of zero charges (pzc) is the pH at which the net charge of the absorbent's surface is zero. The pzc is obtained by acid-base titrations of colloidal dispersions while monitoring the electrophoretic mobility of the particles and pH of the suspension.

### 2.5 Antioxidant activity test

A DPPH radical scavenging assay was conducted to test the ability of ZnO NPs to scavenge free radicals. The NPs' ability to inhibit oxidation was assessed by decolorizing a methanol solution of DPPH. In methanol, DPPH creates a violet or purple color that fades to yellow in the presence of antioxidants. A solution of 0.1 mM DPPH in methanol was mixed with 1 mL of pure ZnO-NPS and ZnO@C-dot in methanol at concentrations of 70, 90, 110, and 130 mg/mL for each nanoparticle. The mixture was vortexed and incubated in the dark at room temperature for 30 min [8]. Using a spectrophotometer, the absorbance of the mixture at 517 nm was measured to determine the percentage of DPPH radical scavenging activity. Ascorbic acid served as the standard.

$$\%\;\text{DPPH}\;\text{radical}\;\text{scavenging}\;\text{activity}=\frac{\left(A_{O}-A_{1}\right)}{A_{0}}X\;100$$

The experiment was repeated three times at each concentration, and the IC50 was determined by plotting the percentage of inhibition against the concentration, with A_0_ representing the control's absorbance and A_1_ representing the sample's absorbance.

### 2.6 Antimicrobial activity

Microbial studies were conducted in Jimma University's Biology Department Laboratory using the agar disc diffusion method [1]. The biological screening effects of synthesized nanoparticles were tested against bacterial strains, including *Staphylococcus aureus*, Salmonella typhus, Bacillus cereus, and *Escherichia coli*. Fungal activity was also tested against Candida albicans. A culture with test tubes of approximately equal concentration or density of 0.5 McFarland standard was used for media inoculation [55 Test bacteria and fungi were seeded on Muller-Hinton Agar and Potato Dextrose Agar, respectively, using freshly grown liquid cultures with similar turbidity to 0.5 McFarland. The negative control was DMSO while Gentamycin and clo were used as positive controls for bacteria and fungi, respectively. An ample solution containing 100 mg/mL of each tested compound was prepared by dissolving them in DMSO. The solutions were then loaded onto the wells of culture and incubated at 37 °C for 24 h. Inhibition zones developed on the plate around the standard paper disc with a diameter of 6 mm, and the antimicrobial activity of nanoparticles was demonstrated by measuring the diameter of the zone of inhibition developed around the sample.

## 3 Result and discussion

### 3.1 Characterization

**Fig. 2:**
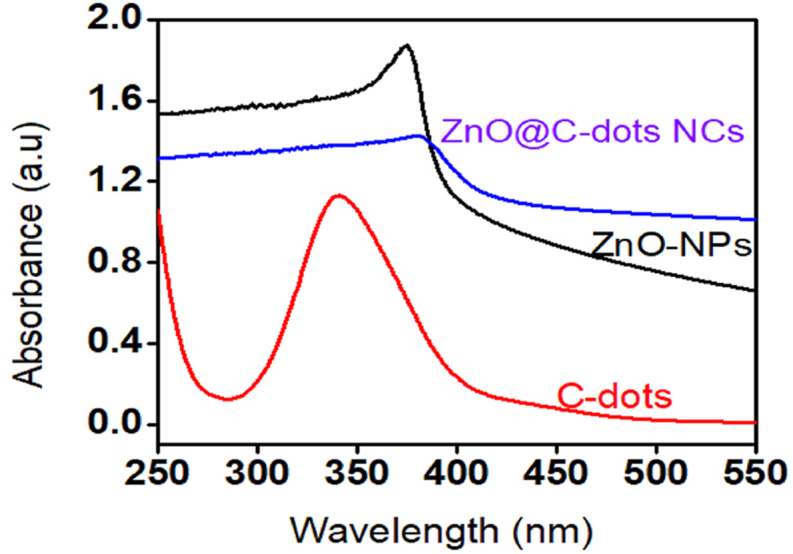
UV–Vis spectrum of C-dots (red) ZnO-NPs (black) ZnO@C-dots Nanocomposite (blue).

UV–Vis, XRD, FTIR and SEM instruments were used for the characterizations of the ZnO-NPs powder and its C-dot nanocomposite obtained by the well-known sol-gel method. Light absorption characteristics of ZnO-NPs and its C-dot nanocomposite (ZnO@C-dot) have been done by spectrophotometer. Fig. 2 shows that the incorporation of C-dot into the pure ZnO-NPs resulted in an absorbance shift towards red. Optimizations of the amount of C-dot required to bring maximum shift were also done. Accordingly, it was found that the incorporation of 10% C-dot (wt/wt) into the pure ZnO-NPs shifted the wavelength of the maximum absorbance from 373 nm to 387 nm. This change is brought on by the surface charge the nanoparticles' improvement and crystallinity.

**Fig. 3:**
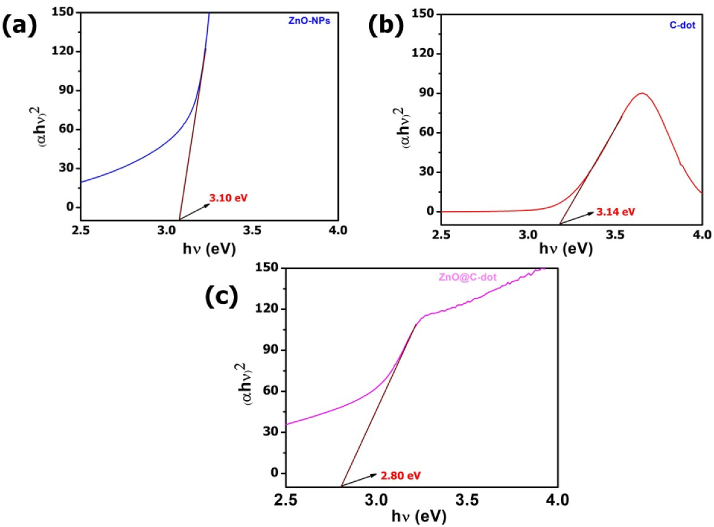
Energy band gaps of a) ZnO NPs, b) C-dots, and c) ZnO@C-dots NCs.

LogA, α, hv, and (αhν^)2^ values were calculated from the UV–vis data to determine band gap energies for all samples. Fig. 3 shows the function of (αhν)^2^ versus photon energy (hv) (for ZnO-NPs, C-dot and ZnO@C-dot. Consequently, the estimated optical band gap of ZnO-NPs shifted from 3.1 eV (Fig. 3a) to 2.8 eV (Fig. 3c)). The reduction of the energy band gap could be attributed to the effect of the incorporation of the C-dot into the pure ZnO-NPs that improved the surface charge and stability of the nanomaterial.

**Fig. 4:**
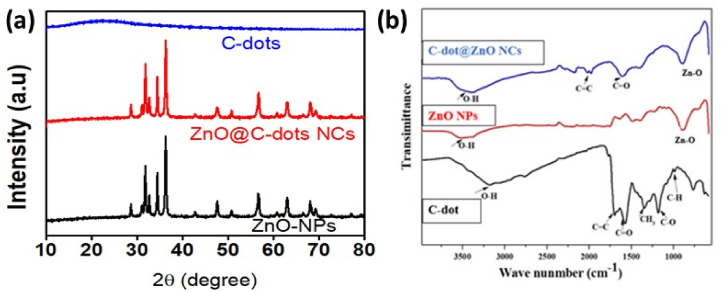
XRD patterns (a) and FTIR spectrum (b) of ZnO-NPs, ZnO@C-dots NCs and C-dots.

The XRD patterns of the pure ZnO-NPs, C-dot, and ZnO@C-dot were measured and shown in Fig. 4a to confirm the crystallinity of the nanoparticles and evaluate the impact of C-dot incorporation on their crystallinity nature. In light of this, the clearly defined peaks were at (2θ) = 29.3, 31.87, 34.36, 36.2, 47.5, 56.53, 62.75, 66.35, 67.85, and 68.99°. The addition of amorphous C-dot materials did not alter the crystalline nature of ZnO-NPs. This is evidenced by the identical diffraction patterns of ZnO@C-dot nanoparticles and hexagonal ZnO (JCPDS card no. 01-080-0074) [28] which are nearly identical to those of pure ZnO. There is no discernible C-dot diffraction peak found in the composite due to the low content and amorphous features of C-dots. The composites show characteristic diffraction peaks similar to those of ZnO nanoparticles, indicating a minimal or no effect of C-dots modification on ZnO crystallinity [29].

FT-IR measurements of as-prepared C-dots and their ZnO nanocomposites were used to compare the chemical structure of the moieties attached to the surface of C-dots and ZnO@C-dots with that of pure ZnO-NPs (Fig. 4b). The nanomaterials' FT-IR spectral bands were recorded between 4000 and 400 cm-^1^. Process parameters for the synthesis of C-dots were also optimized using FT-IR analysis as an indicator. Peaks at 3260 cm^−1^, 1637 cm^−1^, 1371 cm-1, and 1023 cm-1 are observed. Peaks at 480 cm^−1^ and 1084 cm^−1^ correspond to the Zn–O stretching vibration and the bending vibration of the bridging hydroxyl on the surface of ZnO, respectively [29,30]. The surface functional groups in the C-dots are visible in these bands [30,31]. To verify the presence of the surface functionalities and metal-oxygen bonds, FTIR measurements of the pure ZnO-NPs and their C-dot nanocomposite were also conducted. The ZnO@C-dot nanocomposite's FT-IR spectra show distinctive weak bands at 1614 and 1765 cm^−1^ that point to C=C and C=O bonds that were created as a result of the C-dot incorporation and are missing from pure ZnO-NPs**.** Moreover, FT-IR spectra, revealed that the peak intensities of the ZnO@C-dot NCs were decreased when compared with ZnO-NPs, this could be due to the interaction between the ZnO-NPs and C-dots, which results in decreased electron density, confirming the formation of ZnO@C-dot NCs.

**Fig. 5:**
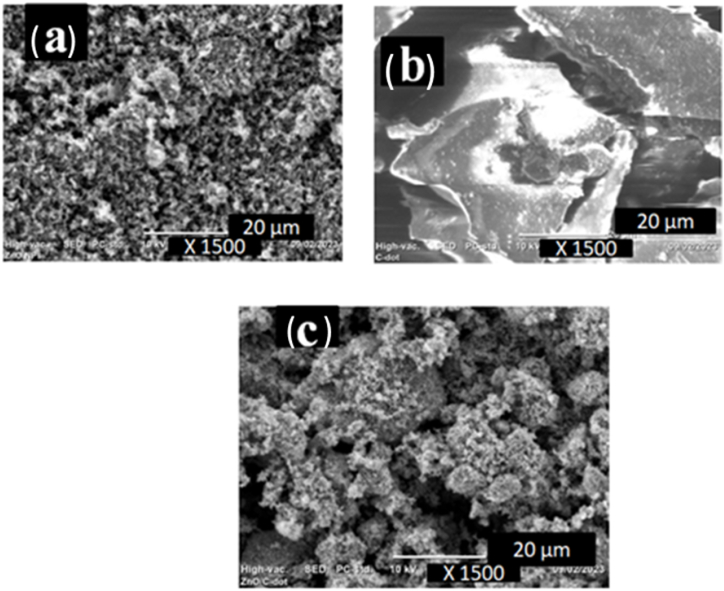
SEM image of a) ZnO NPs, b) C-dots, & c) ZnO@C-dots NCs.

Comparative morphological evaluations of the photocatalyst were also investigated (Fig. 5). The SEM images ZnO-NPs showed that the surface morphologies are in the form of assemblies and cubical distribution over the entire surface as shown (Fig. 5a) which is closely related to the reported kinds of literature [31]. The photocatalyst's surface was enhanced, the cationic dye was better able to adhere to its surface, and the photocatalytic efficiency was increased as a result of the incorporation of the less crystalline C-dot into ZnO-NPs particles. A variety of spherical-shaped, holey, and rod-like structures were seen in the case of ZnO@C-dot NCs, which is nearly similar to the reported literature [32]. As demonstrated in (Fig. 5c), ZnO-NPs show smaller-sized particles while the ZnO@C-dot NCs sample displays relatively larger-sized particles at different magnifications between (1500 and 3000). NPs have a lower size distribution pattern than NCs. This outcome is quite consistent with the XRD data, which demonstrated that ZnO@C-dot NCs formed bigger particle sizes.

### 3.2 Photocatalytic activity

In the current investigation, the photocatalytic degradations of the methyl blue (MB) dye were examined using ZnO@C-dot NCs that were produced using ZnO NPs and C-dots and exposed to sun radiation (@ Jimma) at intervals of 10, 20, 30, 40, 50, and 60 min. The distinctive MB absorption peak strength was discovered at 666 nm and steadily dropped after the dye degradation was visually identified by a gradual shift in the dye solution's color from blue to colorless.

### 3.3 Optimization of photocatalytic degradation of MB dye

**Fig. 6:**
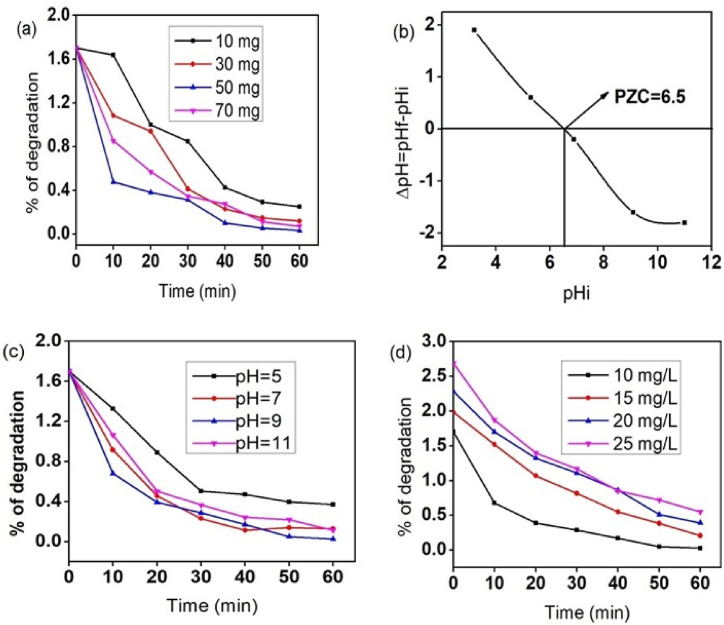
Degradation efficiency of MB on the different parameters a) dosage of catalyst b) point of zero charge c) pH value of solution d) initial concentration of MB.dye under sunlight.

Several photocatalytic requirements, including catalyst dose, Point of Zero Charge, Effect of pH, the Effect of Initial Dye Concentration, and the Effect of Contact Time, were rigorously tuned before the real photocatalytic test, as shown in Fig. 6.

#### 3.3.1 Effect of catalyst dose

From an economic perspective, one of the key elements needed for the degrading effectiveness of pollutants is the amount of photocatalyst. It is important to determine the ideal photocatalyst dose for effective dye removal in order to prevent using too many or too few. As a result, several experiments were carried out to determine the ideal concentration of the photocatalyst (ZnO@C-dot) by adjusting its amount to 10, 30, 50, and 70 mg in a constant initial concentration of MB dye (10 ppm). When the amount of catalyst is changed, the degree of MB dye degradation efficiency is depicted in Fig. 6a. Here, it is important to note that the nanocomposite has more adsorption sites and a bigger surface area when exposed to sunlight for the production of hydroxyl free radicals. These free radicals also have a greater chance of coming into contact with MB dye pollutants. The percentage of MB removed, however, reduced as the photocatalyst dosage was increased further. The increased turbidity of the suspension hinders the photocatalytic process and lowers light penetration [33]. According to Fig. 6a, the degradation of MB rose from 85.2% to 98% when the dose of ZnO@C-dot nanocomposites increased from 10 mg to 50 mg. MB degradation did not significantly increase with dosage increases after 50 mg, though. Therefore, the catalyst dosage of 50 mg was discovered to be ideal for the photocatalytic degradation of 10 ppm MB.

#### 3.3.2 Point of zero charge

In the photocatalytic scheme using ZnO@C-dot nanocomposite, the isoelectric point or point of zero charges (PZC) is reached when the net total particle charge is zero at a specific pH value. During photocatalysis, the PZC of the nanocomposite is an important parameter to demonstrate the variable charge surface of the photocatalyst. This work found the PZC of the ZnO@C-dot nanocomposite in the pH range of 3–11. When the pH of a solution is higher than the PZC value, the composite favors the adsorption of positively charged particles. When the pH value is lower than the PZC, the nanocomposite favors the adsorption of negatively charged dyes (contaminants). Controlling the pH of the photocatalytic system can adjust the surface charge of the photocatalyst, providing the potential for selective degradation of specific dyes. In our case, MB is a well-known cationic dye, and a pH value greater than the PZC value is more favorable for its decomposition (Fig. 6b).

#### 3.3.3 Effect of pH

The adsorption and degradation of the dye depend on the pH of the medium [34]. The rate of degradation in a photocatalytic system depends on the pH level and the adsorption capacity of dyes on the photocatalyst. An increase in the number of target molecules adsorbed on the catalyst results in a higher molecular degradation rate [35]. To study the impact of pH on the efficiency of MB degradation and adsorption capacity, the pH of the MB solution was adjusted to 5, 7, 9, and 11 using 0.1 M HCl and NaOH solutions. The catalyst dosage was 50 mg, and 10 ppm dye was used for 60 min. Fig. 6c shows the plot of percentage degradation at different pH values. It can be observed from the plot that the photocatalytic activity of MB dye on ZnO@C-dot nanocomposite increases with pH up to 9. The highest degradation efficiency was achieved at pH 9, which approximates the pH pzc value of the solution. At acidic pH, the competitive interaction of H+ ion and cationic MB, as well as the repulsive force interaction between the dye cations and the positively charged sites, resulted in lower removal. Conversely, at higher pH, excess hydroxyl groups from the base caused the formation of metal hydroxide complexes, which led to a decrease in MB dye removal. This effect limited the efficiency of synthesized nanocatalysts and reduced the production of free radicals on the surface of the photocatalyst under sunlight radiation. Thus, at 60 min, the maximum removal of methylene blue dye was 98.4% when ZnO@C-dot was added at a pH of 9.

#### 3.3.4 Effect of initial dye concentration

The experiment varied the initial dye concentration (10–25 mg/L) to find the optimal dose. Nano-composites showed higher photocatalytic activity at low dye concentrations (10 mg/L) but decreased efficiency at high dye concentrations (Fig. 6d). This is because when the concentration of dye increases, the solution's color becomes more intense, and the photons' path length entering the solution is reduced. As a result, only a few photons reach the catalyst surface, reducing the production of hydroxyl radicals. The generation of OH• radicals on the catalyst's surface is likely to decrease since the dye ions occupy active sites on the catalyst's surface. The degradation efficiency of MB dye decreased with increased concentration, with the maximum efficiency of 98.4% found at 10 mg/L of MB concentration when ZnO@C-dot was added at a pH of 9. The photodegradation efficiency of 10 mg/L, 15 mg/L, 20 mg/L, and 25 mg/L MB dye at the end of 60 min was 98.4%, 89.4%, 82.9%, and 79.5% respectively. A schematic representation of the mechanism of the dye decomposition is given in Fig. S1.

#### 3.3.5 Effect of Contact Time

**Fig. 7:**
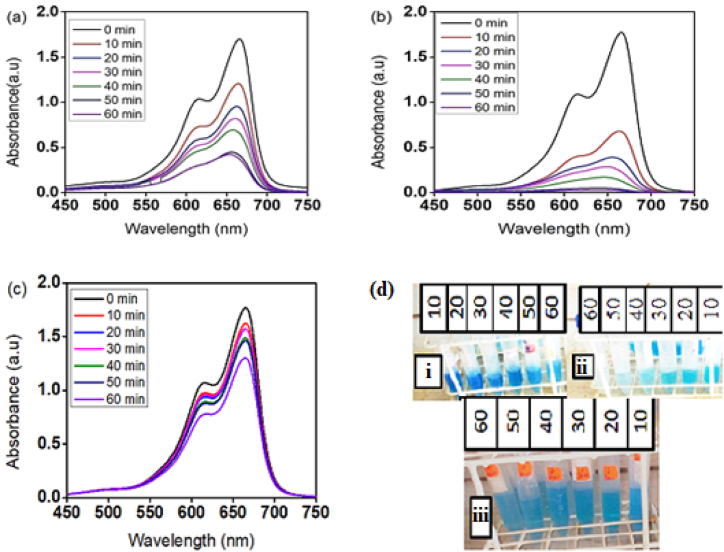
(a–c) UV–Visible absorption spectrum for degradation kinetics of MB dye on a) ZnO-NPs b) ZnO@C-dots NCs and c) photocatalysis under sunlight irradiation (d) Pictures MB at different removal stages with ZnO-NPs (i) & ZnO@C-dot (ii) as the catalyst and without catalyst (iii).

The photocatalytic properties of the prepared ZnO nanoparticles and their C-dot nanocomposites were investigated by decomposing MB. UV–Vis absorbance spectra were investigated to show the impact of irradiation time on the degradation of MB dye and the degradation percentage was calculated by measuring the absorbance. A sample of 20 mL of 10 ppm dye at pH 9 was taken in a volumetric flask and 50 mg of the photocatalyst was added and exposed to light with continuous stirring for 10, 20, 30, 40, 50 and 60-min. After string, the light was irradiated to the three separate systems: ZnO-NPs containing MB, Zn@C-dot containing MB and pure MB: and their UV–vis absorption was measured every 10-min. Fig. 7 shows the UV–Vis absorption spectrum of the degradation kinetics of MB. For the MB containing pure ZnO-NPs the absorbance peaks were gradually decreased upon light irradiation (Fig. 6a). From the UV–Vis measurement, the peak intensity of the MB sample containing ZnO@C-dot drastically decreased upon becoming almost zero after 60-min light irradiation (Fig. 7b). Following the absorbance peak intensity decrease the colour of the solution also faded and became colourless after 60-min light irradiation (Fig. 7d–ii). The maximum degradation efficiency of MB dye was attained by the nanocomposite and 98.4% MB was degraded within 60 min of contact time. On the contrary, MB without the catalyst resisted almost light degradation and peak intensities were almost retained after 60-min light irradiation. The absence of obvious degradation of MB without the photocatalysts (blank test) under light irradiation indicates that the contribution of self-degradation of MB is insignificant and that it resists photodegradation. The slight degradation of MB in pure ZnO-NPs is also attributed to the slow photocatalytic behaviour of ZnO-NPs in visible light due to its high-energy band gap [3,36]. To improve the photocatalytic behavior of ZnO-NPs, doping or forming nanocomposites is important to shift the energy band gap towards the red [[37], [38], [39], [40]]. Incorporating C-dots into pure ZnO-NPs enhances surface charge, and surface area and shifts the band gap to red (Fig. 3c), boosting photocatalytic behavior. As a result, MB with ZnO@C-dot degrades and the blue color disappears in an hour (Fig. 7d).

The radical scavenging properties of the pure doped ZnO NPs and ZnO@C-dot were evaluated through the DPPH method and showed an increased RSA with an increase in the NPs' concentration. The NPs’ half-maximal inhibitory concentration (IC50) was found to be 67.73, 24.40, and 78.40 respectively (Table S2). It was also noted that the ZnO NPs offer high antioxidant activity while ZnO@C-dot has inhibition at 50 μg/mL. This result shows that ZnO@C-dot can prevent oxidation by transferring electron density from oxygen to carbon in DPPH free radical through n→π* transition [41].

**Fig. 8:**
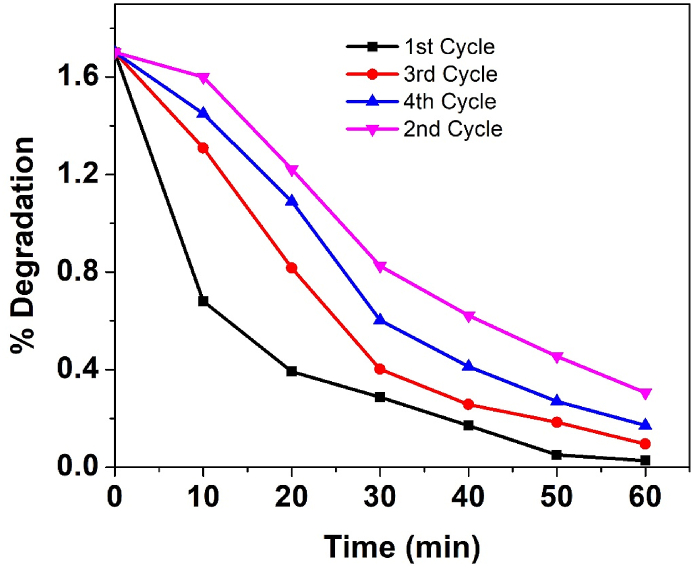
Reusability of ZnO@C-dot using the same optimal operating parameters as we did for the ZnO@C-dot NCs in this experiment, (an initial dye concentration of 10 ppm, a catalyst dose of 50 mg, at a pH of 9, and a 60-min irradiation period.

The ZnO@C-dot composite's recyclability for dye decomposition was determined by the 4-cycle degradation of MB. The photocatalyst was washed with ethanol after each cycle and reused. Fig. 8 shows how many times the ZnO@C-dot NCs photocatalyst can be reused. Using the same optimal operating parameters as the ZnO@C-dot NCs, we performed the reusability test. For the first four cycles, degradation efficiency was 98.4%, 94.4%, 89.9%, and 82.07% respectively at an initial dye concentration of 10 ppm, catalyst dose of 50 mg, pH of 9, and 60-min irradiation period. The photocatalytic degradation efficiency of ZnO@C-dot nanocomposite decreases in the second, third, and fourth cycles, possibly due to the accumulation of waste ions and catalyst dosage leading to impuritiesThis experiment confirms that the synthesized ZnO@C-dot NCs photocatalyst can be recycled for four subsequent cycles with a slight decrease in efficiency. Thus, ZnO@C-dot NCs act as a stable and efficient photocatalyst.

### 3.4 Antimicrobial activity

**Fig. 9:**
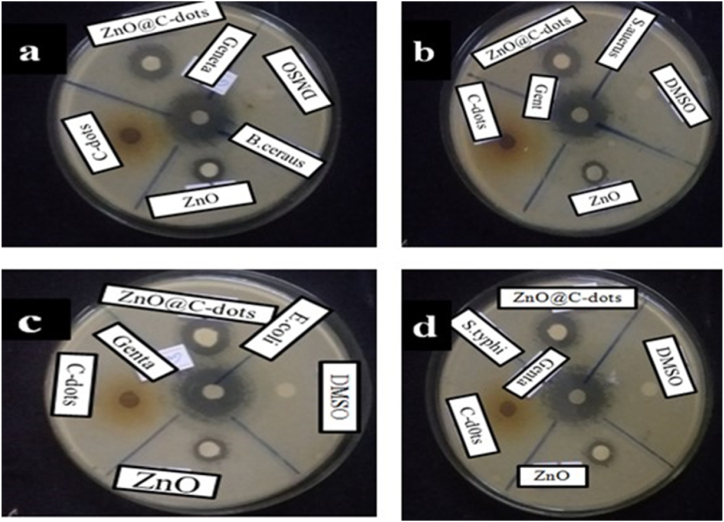
Antimicrobial activity of ZnO-NPs, C-dots, and ZnO@C-dots NCs on (a) B. cereus (b) S.aureus (c) E.coli (d) S. Typhi.

To investigate the antimicrobial activity of synthesized ZnO-NPs, ZnO@C-dot, and C-dot, the disk diffusion method was used against Gram-positive and Gram-negative bacterial strains. Gentamicin and DMSO were used as positive and negative controls, respectively. The antibacterial activities of 25 mg/mL, 50 mg/mL, and 100 mg/mL of synthesized NPs were tested against *S. aureus*, *E. coli*, B. cereus, and *S. typhi*. (Fig. 9a–d). The results of the study showed that gram-positive bacteria, such as *S. aureus* and B. cereus, were more susceptible to the synthesized ZnO-NPs and ZnO@C-dot NCs than gram-negative strains like *E. coli* and *S. typhi*. Additionally, the zinc ions released from the NPs could bind to the negatively charged bacterial cell wall, causing it to rupture, leading to protein denaturation and cell death. Small-sized nanoparticles (NPs) have high penetration ability and cause rapid cell damage compared to other bulk compounds. As NP concentration increases, antibacterial activity improves, consistent with the literature [3]. The nanocomposite's antimicrobial activity was superior to that of pure ZnO-NPs and C-dot, indicating that incorporating C-dot improved the nanocomposite's antimicrobial activity. The inhibition zones of antimicrobial measurements are given in Table 1.

**Table 1:** Microbial Activity of ZnO-NPs, C-dots and ZnO@C-dots nanocomposites.

Microorganism	Inhibition Zone (mm)				
	Controls		Target Material (100 mg/mL)		
	Gentamicin (+ve)	DMSO (-ve)	ZnO-NPs	C-dot	ZnO@C-dot Nanocomposite
B.seraus	24	0	10	9	15
S.aureus	23	0	13	9	16
E.coli	25	0	12	8	15
S.typhi	24	0	10	7	14

## 4 Conclusion

ZnO nanoparticles and their C-dot nanocomposites were synthesized using the co-precipitation method with aqueous precursor solutions. We analyzed the crystal structure, band gap energy, bond vibration properties, and morphologies of the photocatalysts using XRD, double beam UV–Vis, FT-IR, and SEM. Furthermore, the addition of C-dot to ZnO-NPs enhanced surface charge and uniformity, as indicated by FTIR analysis. This also led to a decrease in the energy bandgap of ZnO-NPs from 3.1 to 2.8 eV. The synthesized nanoparticles and nanocomposite-treated MB dyes were under visible light to evaluate their photocatalytic activity. As a result, MB dye was successfully decolourized by the ZnO@C-dot photocatalyst under direct solar irradiation was observed from pH PZE and FT-IR measurements that the addition of C-dot materials enhanced the surface charges of the nanocomposites, resulting in improved photocatalytic degradation efficiency. Due to the improved surface charge, the photocatalytic degradation efficiency of ZnO@C-dot surpassed that of pure ZnO-NPs. ZnO@C-dot prepared at pH 9 achieved an optimum efficiency of 98.4% degradation. This approach can enhance the photocatalytic properties of various nano photocatalysts.

## Author contributions

Conceived and designed the experiments: Asegid Belete Tegenaw, Ahmed Awol Yimer, Tamene Tadesse Beyene; Performed the experiments: Asegid Belete Tegenaw, Ahmed Awol Yimer, Tamene Tadesse Beyene; Analyzed and interpreted data: Asegid Belete Tegenaw, Ahmed Awol Yimer, Tamene Tadesse Beyene; Wrote the paper: Asegid Belete Tegenaw, Tamene Tadesse Beyene; Contributed Reagents, Materials, Analysis Tools or Data; Tamene Tadesse Beyene.

## Declaration of competing interest

The authors declare that they have no known competing financial interests or personal relationships that could have appeared to influence the work reported in this paper.
