# Supplementary material for: Boosting the photocatalytic activity of ZnO-NPs through the incorporation of C-dot and preparation of nanocomposite materials
Source: Heliyon. 2023 Oct 5;9(10):e20717. doi: 10.1016/j.heliyon.2023.e20717 (PMC10570576; doi:10.1016/j.heliyon.2023.e20717)
Supplement: Multimedia component 1 [file mmc1.docx]

**Boosting the Photocatalytic Activity of ZnO-NPs through the Incorporation of C-dot and Preparation of Nanocomposite Materials**

**Asegid Belete Tegenaw ^a^, Ahmed Awol Yimer ^a^ and Tamene Tadesse Beyene,*^, a^**

a Department of Chemistry, College of Natural Sciences, Jimma University, P.O.Box 378, Jimma-Ethiopia;

* Corresponding Author

E-Mail: [tamene.tadesse@ju.edu.et](mailto:tamene.tadesse@ju.edu.et)

| **Time interval(sec)** | **Ib (W/m^2^)** | **m(kg/s)** | **QL(W)** | **Qu(W)** | **Qabs (W)** | **QS(W)** | **EFFth (%)** | **Ib (W/m2)** |
| --- | --- | --- | --- | --- | --- | --- | --- | --- |
| 12:00 PM | 834.12 | 0.004950495 | 287.95 | 931.19 | 1219.36 | 2122.58 | 0.45 | 0.004950495 |
| 12:30 PM | 813.87 | 0.004901961 | 281.19 | 942.55 | 1203.47 | 2071.05 | 0.47 | 0.004901961 |
| 13:00 PM | 797.00 | 0.004166667 | 252.68 | 792.46 | 1036.62 | 2028.12 | 0.41 | 0.004166667 |
| 13:30 PM | 732.96 | 0.003703704 | 195.83 | 696.67 | 892.67 | 1865.15 | 0.39 | 0.003703704 |
| 14:00 PM | 656.21 | 0.002890173 | 173.80 | 543.64 | 717.57 | 1669.85 | 0.34 | 0.002890173 |

**Table S1**: *Average System thermal calculation for January (at the time of investigation)*

**Table S2**: *DDPH radical scavenging assay*

| **Materials** | **Concentration (mg/mL)** | **%RSC of DDPH** | **IC50 (mg/mL)** |
| --- | --- | --- | --- |
| ZnO-NPs | 70 | 40.98380181 | 67.73840609 |
|  | 90 | 48.40400191 |  |
|  | 110 | 50.04764173 |  |
|  | 130 | 52.45354931 |  |
| Zn@C-dot | 70 | 65.895664602 | \| 24.40025434 \| \| --- \| \| \| |
|  | 90 | 71.20771796 |  |
|  | 110 | 81.89947594 |  |
|  | 130 | 88.94949976 |  |
| Ascorbic acid | 70 | 97.6680324 | \| 43.75954198 \| \| --- \| \| \| |
|  | 90 | 98.70152454 |  |
|  | 110 | 98.8325393 |  |
|  | 130 | 98.9231062 |  |

**Table S2:** *Comparative summary of the current work with the previously reported literatures.*

| ***Material*** | ***Catalyst Dose***  ***(mg)*** | ***Pollutant*** | ***Time***  ***(Minute)*** | ***Degradation Efficiency (%)*** | ***Reference*** |
| --- | --- | --- | --- | --- | --- |
| *Cu-doped ZnO-NPs* | *70* | *MB* | *120* | *85.00%* | *[1]*  [*https://doi.org/10.1155/2022/9459886*](https://doi.org/10.1155/2022/9459886) |
| *ZnO-NPs* | *10* | *MB* | *90* | *89.12%* | *[2]*  [*https://doi.org/10.1039/D2RA06967A*](https://doi.org/10.1039/D2RA06967A) |
| *Ag-doped ZnO-NPs* | *-* | *MB* | *80* | *96.00%* | *[3]*  [*https://doi.org/10.1038/s41598-020-77426-y*](https://doi.org/10.1038/s41598-020-77426-y) |
| *ZnO–SnO_2_–Sn Nanocomposite* | *-* | *MB* | *-* | *95.60%* | *[4]*  [*https://doi.org/10.11648/j.am.20160504.12*](https://doi.org/10.11648/j.am.20160504.12) |
| *NiO-NPs* | *-* | *MB* | *150* | *<50%* | *[5]*  [*https://doi.org/10.1016/j.matpr.2017.09.083*](https://doi.org/10.1016/j.matpr.2017.09.083) |
| *CeO-NPs* | *-* | *MB* | *-* | *~90%* | *[6]*  [*https://doi.org/10.1021/acsomega.3c00198*](https://doi.org/10.1021/acsomega.3c00198) |
| *ZnO@C-dot* | *50* | *MB* | *60* | *98.4%* | *This Work* |


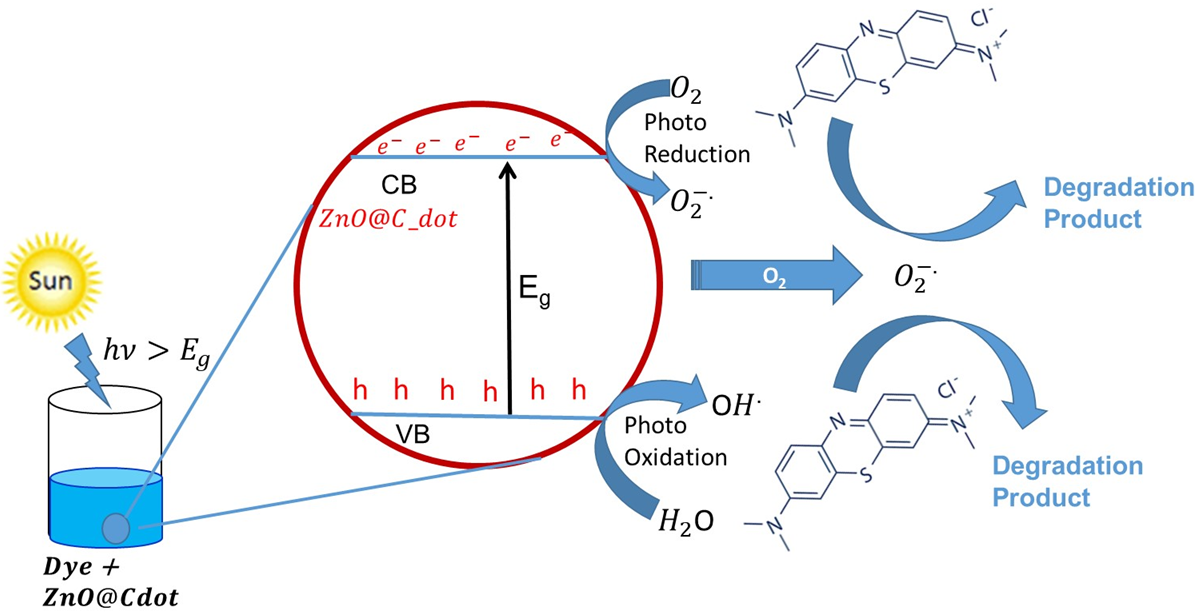


**Figure S1**: Schematic representation of the mechanism of methylene blue decomposition.

**Reference**

1. Khalid, A., et al., *Effect of Cu Doping on ZnO Nanoparticles as a Photocatalyst for the Removal of Organic Wastewater.* Bioinorganic Chemistry and Applications, 2022. **2022**: p. 9459886.

2. K K, S., P.N. P M, and M. Vasundhara, *Enhanced photocatalytic activity in ZnO nanoparticles developed using novel Lepidagathis ananthapuramensis leaf extract.* RSC Advances, 2023. **13**(3): p. 1497-1515.

3. Alharthi, F.A., et al., *Facile one-pot green synthesis of Ag–ZnO Nanocomposites using potato peeland their Ag concentration dependent photocatalytic properties.* Scientific Reports, 2020. **10**(1): p. 20229.

4. Długosz, O. and M. Banach, *ZnO–SnO2–Sn nanocomposite as photocatalyst in ultraviolet and visible light.* Applied Nanoscience, 2021. **11**(5): p. 1707-1719.

5. Jayakumar, G., A. Albert Irudayaraj, and A. Dhayal Raj, *Photocatalytic Degradation of Methylene Blue by Nickel Oxide Nanoparticles.* Materials Today: Proceedings, 2017. **4**(11, Part 3): p. 11690-11695.

6. Kalaycıoğlu, Z., et al., *Efficient Photocatalytic Degradation of Methylene Blue Dye from Aqueous Solution with Cerium Oxide Nanoparticles and Graphene Oxide-Doped Polyacrylamide.* ACS Omega, 2023. **8**(14): p. 13004-13015.

7. Talib, R., et al., *ZnO Nanorods/Polyaniline-Based Inorganic/Organic Heterojunctions for Enhanced Light Sensing Applications.* ECS Journal of Solid State Science and Technology, 2016. **5**: p. P1-P6.
